# Plant Temperature Sensors

**DOI:** 10.3390/s18124365

**Published:** 2018-12-10

**Authors:** Tomoaki Sakamoto, Seisuke Kimura

**Affiliations:** 1 Department of Bioresource and Environmental Sciences, Kyoto Sangyo University, Kamigamo Motoyama Kitaku, Kyoto 603-8555, Japan; k5774@cc.kyoto-su.ac.jp; 2 Center for Ecological Evolutionary Developmental Biology, Kyoto Sangyo University, Kamigamo Motoyama Kitaku, Kyoto 603-8555, Japan

**Keywords:** temperature sensor, phytochrome, heat shock transcription factor A1s

## Abstract

Temperature is one of the most important environmental signals for plants. High and low temperatures have a variety of effects that affect plant growth and development profoundly. Further, temperature is an indication of seasonal change. Plants must survive under severe conditions in winter and prepare to resume growth and reach their reproductive stage in the following spring. Recent studies have focused on plant mechanisms responsible for sensing temperature and the molecular systems underlying plant reactions in response to this signal. In this review, we describe how plants sense ambient temperature to adapt to ambient-temperature changes.

## 1. Temperature and Living Organisms

Living organisms can be found in widespread regions of the world. Each habitat consists of a certain set of environmental factors, among which one of the most important for any organism is ambient temperature. In some habitats, temperatures may be higher than 40 °C or lower than freezing point. Temperatures outside the narrow range within which a given living organism finds itself comfortable are not suitable for successful growth and reproduction and, indeed, might be harmful and even limiting to survival. Temperature affects different biological phenomena in living organisms, for example, enzyme activity is markedly temperature dependent, and molecular processes working inside organisms are regulated by enzymes that show optimal temperature ranges for best performance. Homeotherms, such as mammals, keep body temperature within a narrow optimal range through the production of heat inside their body, and by a set of thermoregulation mechanisms. Other animals move away in an attempt to escape severe environmental temperature changes. As this is impossible for plants, they do their best to adapt to temperature change by modulating their physiological function in response to temperature changes. Temperature also acts as a seasonal signal for plants. In temperate zones, temperature and other environmental factors change seasonally. Because some seasonal conditions are not favorable for plants, they stop growing and do not reproduce for prolonged periods while severe conditions prevail, and adjust so as to grow in the preferred condition. Some systems of plant adaptation use regular, periodical change in ambient temperature as a cue for sensing seasonal change.

The study of the plant response systems to temperature change has revealed the underlying molecular mechanisms. Here, we review the plant temperature response systems and the core mechanisms of temperature sensors.

## 2. Effects of Temperature on Growth and Development

Plants have systems that regulate their internal physiological reactions according to ambient temperature. As a result, plants sometimes show modulation of growth and morphological changes in response to temperature changes.

### 2.1. Tissue Elongation under High Temperature

Light condition affects tissue size. In the dark, seedlings grow long hypocotyls and etiolated cotyledons, like soybean sprouts. High temperature also causes tissue elongation. For example, the hypocotyl of *Arabidopsis thaliana* elongated at high temperature even in the presence of light [1]. Leaf petiole elongation and leaf hyponasty responses were also studied in *A. thaliana* under high temperature [2]. The hyponastic response consists of an upward bending of the petiole by increased cell elongation on their lower longitudinal side. At low temperature leaves grew horizontally, while at high temperature the angle of leaf turned upright.

### 2.2. Leaf Shape Modification

Leaf shape is another aspect of plant development affected by temperature. Some plants show a change in leaf shape induced by an environmental cue; this phenomenon is called heterophylly. *Rorippa aquatica*, a semi-aquatic plant that lives both on land and in water, grows leaves with different shapes under different growing conditions. When submerged, the leaves become narrow and highly dissected, compared to those grown on land. Furthermore, under high temperature the plant developed round simple leaves, while low temperature induced dissected compound leaves [3]. Leaves of red maple trees (*Acer rubrum*) grown under cooler temperatures tended to have more teeth on the leaf edge and to be more highly dissected [4].

A similar phenomenon was studied in the pitcher plant, *Cephalotus follicularis* [5]. In addition to normal flat leaves, this carnivorous plant is provided with pitcher leaves to capture insects. The ratio of pitcher to flat leaves varied depending on temperature. Most leaves were flat at 15 °C, while about 90% of them developed into pitcher leaves at 25 °C.

### 2.3. Thermotolerance

For any given plant species, temperatures above physiological range eventually cause cellular metabolic imbalance, thereby interfering with growth and reproductive development, and eventually lead to death. Thermotolerance is the resistance to high temperature. Plants have evolved both an inherent resistance to high temperature (basal thermotolerance) and an ability to activate tolerance in response to heat stress (acquired thermotolerance). Acquired thermotolerance was induced by either exposure to a short period of high temperature [6,7] or a gradual temperature increase [6]. These results showed that plants positively prepare for prolonged heat conditions by activating the heat-shock response pathway.

## 3. Temperature as a Seasonal Cue

Day length and temperature vary annually, especially in temperate zones. Extreme low temperature in winter is severe for plant growth and reproduction. Plants use temperature as a seasonal cue. The accumulation of the temperature signal acts as a seasonal signal that determines the timing of crucial physiological events.

### 3.1. Flowering Time

In some plant species, the timing of flowering is regulated by temperature. Based on several research data sets, Jagadish et al. [8] reported a trend for earlier flowering in many plant species in response to increased temperature under natural conditions, beginning with observations made as early as the 19th century. Effects of temperature on flowering have been examined under experimental conditions. In *Arabidopsis*, wild-type strain Landsberg erecta (Ler) flowered when the total leaf number reached about 40 under short day conditions at 23 °C; on the other hand, it flowered when the leaf number reached 15 at 27 °C [9]. Similarly, rice plants grown at 23 °C bloomed later than at 27 °C [10]. In these plant species, high temperature accelerated flowering. However, this does not apply to all plant species. For example, high temperature delayed flowering of *Chrysanthemum morifolium* and *C. seticuspe* [11].

### 3.2. Vernalization

Vernalization is one of the long-term seasonal effects of temperature. It is the phenomenon whereby flowering is induced by exposure to a relatively long period of cold. Subsequently, flowering initiates upon exposure to favorable conditions in the following growing season. Vernalization was first reported as a treatment of winter wheat planted in autumn to ensure abundant flowering the following spring. Vernalization is a widespread trait in plants of temperate zones. The pathway has been studied in several plant species and was recently reviewed [12]. The following steps have been elucidated: A suppression of flowering before winter and achievement of competence to flower by cold via the suppression of a floral repressor (e.g., *Arabidopsis*) or the activation of a floral promoter (e.g., Pooideae).

## 4. The Mechanisms Sensing Temperature

As described above, temperature is an important environmental factor for plants. To respond to temperature changes, plant tissues must have the way to sense it. The sensory systems have been studied since these temperature dependent responses were first observed. Recent studies have focused on identifying the elements comprising such sensory systems. However, only a part of the temperature sensory systems was identified, and unknown temperature sensors might still exist. In this section, we note the identified mechanism whereby plants sense temperature and its primary signal transduction.

### 4.1. Phytochrome B

**Figure 1:**
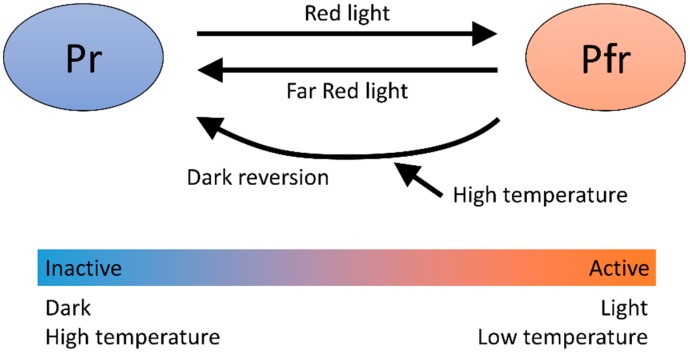
Light and temperature regulation of phyB activity. Phytochrome B state changes in response to light and temperature. It is activated by light, while it is inactivated in the dark and by high temperature through acceleration of dark reversion.

One of the temperature sensors best established and analyzed is phytochrome B (phyB). Phytochrome B is the gene that encodes a light-sensing protein present in two alternative forms, depending on intensity and quality of light [13,14] (Figure 1). The inactive form of phyB, which is called Pr, absorbs maximally at the red wavelength of the electromagnetic spectrum. When phyB Pr is exposed to red light, it changes to the active Pfr form. Active phyB Pfr shows maximum absorbance in the far-red region of the spectrum. Far-red radiation accelerates the rate at which Pfr converts to Pr. The phyB Pfr spontaneously relaxed into Pr independent of light. This phenomenon is called dark or thermal reversion. Under natural conditions, sunlight activates phyB to the Pfr form and phyB becomes inactive at night.

Some temperature-mediated responses (elongation of hypocotyl and petiole, and leaf hyponasty) are similar to shade-avoidance responses, which occur under shade to escape from the shade of a tree canopy. This similarity suggested that responses to high temperature and light–dark transitions likely share the same molecular pathway. Under shade, light quality differs from direct sunlight. The ratio of red to far-red (R/FR) decreases since plants absorb red and blue light. Phytochrome B is the main photoreceptor controlling tissue elongation under shade conditions [15]. In a recent study, it was identified that phyB acts as temperature sensor through temperature-dependent reversion of phyB. The measurement of the half-life of active state phyB (Pfr) in vitro [16] and in vivo [17] showed that reversion from Pfr to Pr proceeded faster at higher temperature. This indicated that phyB became more inactive at a higher temperature if the light condition was the same. Thus, phyB acts as a temperature sensor through the interconversion between active and inactive states in response to temperature.

**Figure 2:**
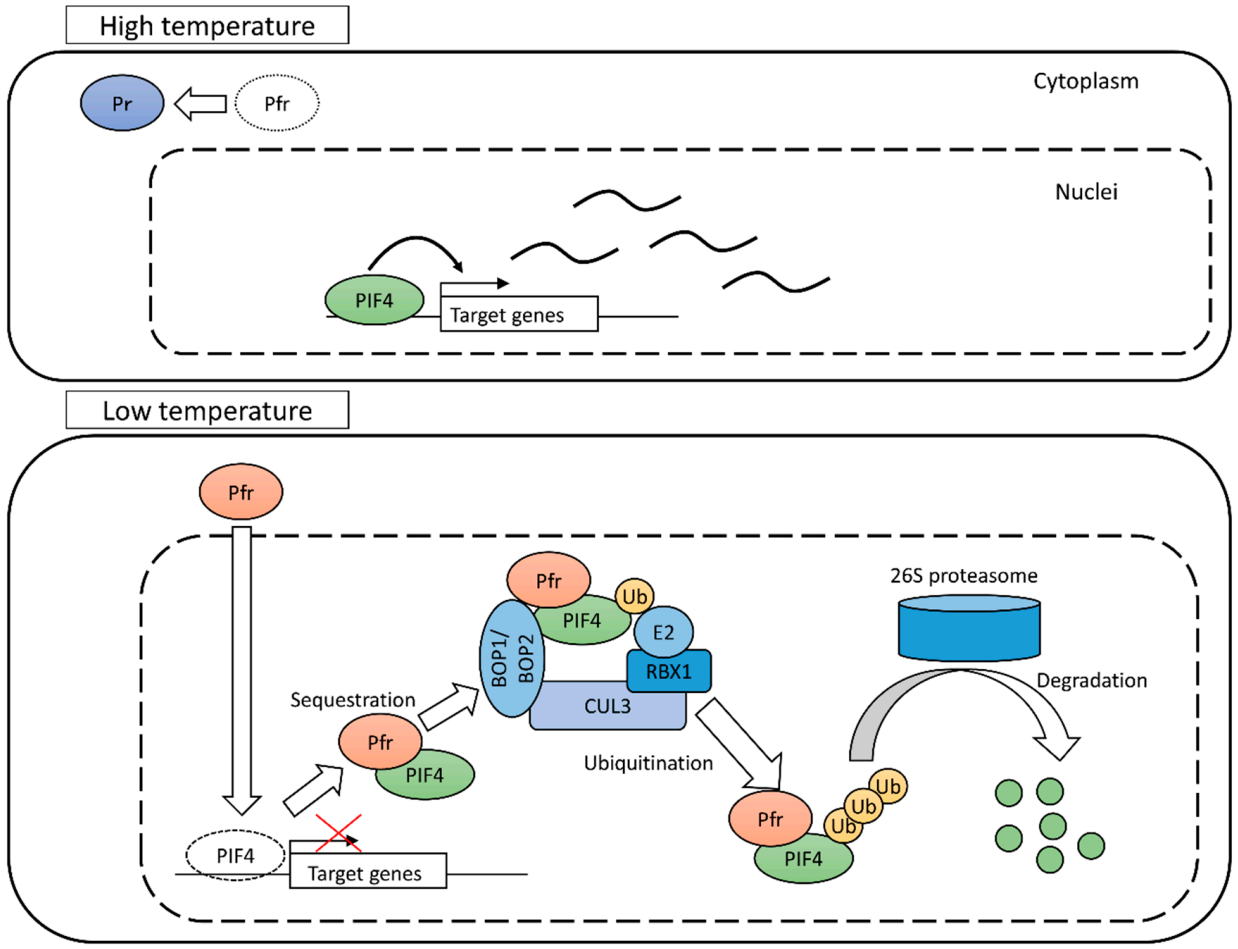
The mechanism of the temperature sensing system based on phyB and PIF4. At high temperature, phyB gets converted to the inactive Pr state and is located in the cytoplasm. The PIF4 binds to the regulatory region of target genes and regulates their expression. At low temperature, phyB is in an active state and enters the cell nucleus. The PIF4 is sequestrated from target genes by binding to phyB. The PIF4 is degraded by 26 proteasomes through ubiquitination. The activity of PIF4 is suppressed at low temperature.

Phytochrome B might work as a temperature sensor by the conversion of its states. It must output the temperature signal to regulate various physiological responses. Phytochromes can interact with other proteins called Phytochrome Interacting Factors (PIFs) [18,19]. These are the genes encoding transcriptional factor proteins which have the basic helix–loop–helix (bHLH) DNA binding domain. Transcriptional factor proteins enter the cell nucleus and bind to a specific region of genomic DNA and regulate the transcription of target genes. In *Arabidopsis*, *PIF1* (also named *PIF3-LIKE 5* or *PIL5*), *PIF3*, *PIF4*, *PIF5* (*PIL6*) and *PIF7* belong to the *PIF* gene family. In this family, *PIF4* might be most essential for temperature-dependent responses. Loss of function mutant of *PIF4* did not show hypocotyl elongation under high temperature, while other tested mutants of *PIF3* and *PIF5* showed hypocotyl elongation similar to the wild type [2]. The mechanism whereby phyB regulates *PIF4* activity has been elucidated by the study of light-dependent responses. It consists of two different strategies: Degradation and sequestration (Figure 2).

An experiment using a transformant in which *PIF4* was constitutively expressed showed that PIF4 protein was degraded in response to red light and re-accumulated under dark conditions [20]. A similar phenomenon was reported for the temperature response. The PIF4 protein was more stable at 25 °C than at 15 °C [21]. In the experiment using PIF4 conjugated-green-fluorescent protein, PIF4 abundance increased more at 28 °C than at 22 °C, especially in nuclei [22]. These results suggest that the system positively degraded PIF4 under red light and low temperature. The mechanisms of degradation consist of ubiquitination of the PIF4 protein and subsequent degradation by 26S proteasomes. Ubiquitination serves to label proteins for degradation. Ubiquitinated proteins are transferred to 26S proteasomes, which are sites of protein degradation. In a very recent study, it was revealed how PIF4 was ubiquitinated [23]. Ubiquitination of PIFs is executed by the CUL3-based E3 ligase complexes composed of a CUL3 backbone, an E2-Ub-docking RING Box1 (RBX1) protein and a member from BTB-domain containing protein family. A BTB-domain containing protein acts as a target-substrate recognition component [24]. The kind of BTB-domain containing protein in complex decides which protein is ubiquitinated. For degradation of PIF4, two BLADE ON PETIOLE proteins (BOP1 and BOP2) in the BTB-ankyrin protein family are related to reorganization of PIF4. The ubiquitination and degradation of PIF4 are reduced in the *bop1 bop2* mutant background, compared with the wild type not only under red light but also under elevated temperature. It is suggested that CUL3-based E3 ligase complexes containing BOP proteins control PIF4 protein abundance under red light as well as under elevated temperature.

Phytochrome B also controls PIF4 activity by sequestration. The site where PIF4 acts to regulate gene expression is the cell nucleus. The PIF4 binds expression-regulatory regions of target genes in genomic DNA thorough its bHLH domain. Localization of PIF4 in nuclei is an essential condition for their activity. The phyB Pfr binds to PIFs thorough an Active phyB-Binding (APB) domain. After binding to PIFs in the nucleus, this complex is removed from the binding site on genomic sequence. In an experiment using a phyB mutant without degradation activity, the expression of genes promoted by PIFs were reduced under red light conditions [25]. This result indicated that active phyB can repress PIF activity by sequestration without degradation.

The repression of PIF activity was confirmed as an exclusive response to light that might vary depending on temperature. Eventually temperature signal in this pathway is output through the change of transcriptional regulation activity of PIF4. The PIF4 up-regulates expression of the genes related to biosynthesis of auxin, which is a kind of phytohormone and control growth and development [26,27].

### 4.2. Temperature Sensing for Heat Shock Response

Heat shock transcription factorA1s (HSFA1s) belongs to a cluster of critical factors in the heat shock response. Knockdown or multiple knockout mutations of HSFA1 genes in tomato and *Arabidopsis* cause the reduction of thermotolerance [28,29]. HSFA1s encodes a master transcriptional regulator of downstream heat-shock responsive genes. The regulation of their activities in response to heat stress is central to the mechanisms underlying heat shock responses. Phosphorylation/dephosphorylation and protein–protein interactions control HSFA1s activity. The HSFA1s protein is phosphorylated by calmodulin-binding protein kinase 3 (CBK3) [30] and dephosphorylated by protein phosphatase 7 (PP7) [31]. The fact that cbk3 and pp7 mutants show less thermotolerance led us to conclude that the modulation of the phosphorylation status of HSFA1s by these genes has an important role in heat-stress response through HSFA1s. However, phosphorylation/dephosphorylation sites and the mechanisms that regulate phosphorylation activity in response to high temperature are not clear.

Another mechanism for the regulation of HSFA1s involves protein–protein interactions, for example, Heat Shock Protein 70 (HSP70) and HSP90. They are up-regulated by heat stress, although they exist under normal conditions, in which they bind to HSFA1s to repress its nuclear localization and subsequent transcriptional activation [32,33]; when plants are subjected to heat stress, HSFA1s protein is released by HSP70 and HSP90, thereby becoming active in triggering the heat responsive cascade [34].

### 4.3. Other Temperature Sensing Systems

**Figure 3:**
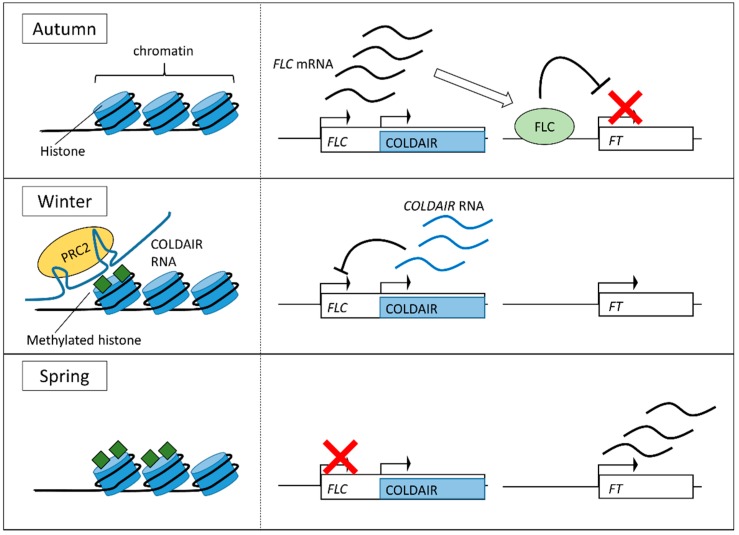
The mechanism of the vernalization system. The diagram shows chromatin state near the *FLOWERING LOCUS C* (*FLC*) gene (left), *FLC* and COLDAIR regions and their expression (center) and *FT* region (right). In autumn or under conditions which did not include a prolonged cold period, the *FLC* gene is expressed and the FLC protein suppresses the expression of *FLOWERING LOCUS T* (*FT*). Conversely, in winter, COLDAIR noncoding RNA is transcribed from the 1st intron of *FLC*. COLDAIR RNA binds to Polycomb Repressive Complex 2 (PRC2) and guides it to the *FLC* region in the chromosome. Polycomb Repressive Complex 2 starts methylation of histones in the *FLC* region and this methylation deactivates the expression of *FLC*. In the following spring, expression of COLDAIR stops, but *FLC* still remains suppressed. The *FT* is activated and then differentiation into flowering begins.

The mechanism of vernalization was analyzed using *Arabidopsis*, in which a floral repressor *FLOWERING LOCUS C* (*FLC*) represses differentiation into floral stage by suppression of *FLOWERING LOCUS T* (*FT*), which is a component of florigen, the flowering inducing factor [35,36] (Figure 3). This suppression was overcome by COLDAIR, which is a long noncoding RNA transcribed from the first intron of *FLC* [37]. COLDAIR started to express after transition to cold temperature and its expression peaked 20 days later, decreasing thereafter even under prolonged cold conditions. COLDAIR RNA formed secondary structures and bound to Polycomb Repressive Complex 2 (PRC2). This is one of the chromatin-remodeling complexes that regulate transcriptional activity by modulating chromatin state of target genes in the genome [38,39]. COLDAIR-PRC2 complex bound to *FLC* region and suppressed *FLC* expression through conversion of chromatin into the inactive state through histone methylation [40]. As a result of the suppression of *FLC*, the expression of *FT* and other flowering-regulating genes were able to activate in a subsequent warm period, thereby initiating flowering-associated differentiation. Although the pathway that promotes flowering by the cold signal was identified, the regulation of COLDAIR expression and chromatin modification in response to low temperature is not clear. Temperature sensing components that are not yet known might be involved in this pathway.

Another component of thermo-sensory responses is H2A.Z, a variant of H2A, which is a kind of histone and a component of the nucleosome. An *ARP6* mutant involved in the activity of H2A.Z histone substitution in place of H2A revealed various traits responding to temperature: Hypocotyl elongation, petiole elongation and acceleration of flowering. Increased occupancy by H2A.Z near the transcription start-site prevents expression of that gene. Temperature dependent variation of H2A.Z occupancy regulated the expression of temperature responsive genes [41]. In further analysis, Kumar et al. [42] showed that H2A.Z-nucleosomes were present at the *FT* promoter at 17 °C and the levels of H2A.Z-nucleosomes at the *FT* promoter decrease under higher temperature (27 °C). In a recent study, it was found that H2A.Z-nucleosomes occupancy and the depletion of them in response to high temperature are enriched at temperature responsive genes regulated by HSFA1 [43]. These results suggest that H2A.Z is important for various temperature mediated responses. However, how to convert H2A.Z level in response to temperature is still unknown.

Involvements of other photoreceptors in temperature responses were shown in a recent study. Although high temperature induces elongation of hypocotyl as described above, radiation of blue light inhibits the high temperature mediated hypocotyl elongation and blue-light receptors Cryptochrome 1 (CRY1) is essential for this inhibition [44]. A similar phenomenon was found in relation to ultraviolet-B light (UV-B). Ultraviolet-B light also represses high temperature mediated elongation of hypocotyl and petiole. Ultraviolet-B light photoreceptor UV RESISTANCE LOCUS 8 (UVR8) is relevant to this inhibition [45]. These photosensors might be involved in temperature response through PIF4 by regulation of E3 ubiquitin ligase activity of COP1 (CONSTITUTIVE PHOTOMORPHOGENIC 1) [44,45]. In another case of involvement of photoreceptor, blue light photoreceptor phototropin might act as temperature sensor. Chloroplasts change their intracellular position in response to light and temperature conditions. Phototropin has an important role in sensing blue light and temperature for this response. The temperature-sensing mechanism of phototropin might be similar to that of phyB. The lifetime of activated phototropin by blue light became longer at low temperature [46].

## 5. Conclusions

Evidence shows that plants possess various types of temperature-sensing systems, some of which have been identified and, at least partially, characterized in terms of composition and operation. In this review, we have discussed only the core aspect of temperature sensing and the response systems involved. As a whole, all of these systems, including related pathways and downstream reactions, are highly complex and have not been thoroughly elucidated.

Plants have multiple temperature sensors to adapt to various aspects of temperature effect. Although both phyB/PIF4 pathway and heat shock response are able to respond to short-term temperature change, the responsiveness of each one is different. Small temperature elevation causes morphological change in phyB/PIF4 pathway. On the other hand, it acts as a cue for following heat stress in heat shock response [43].

Although temperature is an important environmental signal, it is not independent from other environmental factors under natural conditions; indeed, it is tightly linked to other factors. Light, in particular, is closely related to temperature. A decrease in light irradiation causes temperature to drop in winter, whereas an increase causes temperature to rise in spring. In plants, sensing systems for each environmental factor might actively crosstalk to each other. The activity of phyB is regulated by both light and temperature, and other photosensors are also related in temperature-mediated responses. The heat shock response pathway enhances the resistance to drought, as well as heat stress [47]. The response system for each environmental factor might also share downstream pathways. Plant growth and development are controlled by phytohormones (auxins, gibberellins and others), and several response systems are based on regulation of these phytohormones. Even in more upstream hormone regulation, temperature response systems are affected by other pathways. PhyB shares the transcriptional regulation pathway of PIF4 with Cry1 and UVR8. The phyB signal pathway and circadian clock signal are integrated through regulation of *PIF4*. A component of circadian clock, Evening Complex (EC) bound to *PIF4* promoter and repressed its expression [48,49]. High temperature reduced the binding of EC to *PIF4* promoter and thereby the repression of *PIF4* was released [49]. These results suggested that *PIF4* was also regulated on a transcriptional level dependent on temperature through circadian clock signal pathway. The relationship between EC and phyB was studied. Evening Complex and phyB showed protein–protein interaction [50] and co-binding to transcriptional regulatory region of target genes [49]. Multiple environmental signals were integrated and output through these signal pathways. The expression of HSP70 is controlled by both heat shock response pathway and light-induced chloroplast signaling. The gating by light-induced chloroplast signaling limits heat shock gene expression to the daytime [51]. Although temperature sensors consist of simple components, whole pathways for responding to temperature are complex.

Further, temperature-response systems show downstream feedback-regulation. The phyB-PIF4 pathway for control of phytohormones provides a clear illustration. A kind of phytohormones, brassinosteroid was up-regulated by PIF4, whereby the brassinosteroid responsive gene enhanced the expression of PIF4 [52]. They formed a positive feedback loop. In the heat-stress pathway, the amounts of HSP70 and HSP90 increased in response to high temperature [33]. This increase might have affected the interaction between HSPs and HSFA1s. This relation suggested that the sensory system was affected by feedback from downstream through these HSPs.

The study of various temperature-response systems has resulted in the identification of some of the temperature sensors involved in each case. However, the details of the molecular mechanisms that sense temperature in plants are still unknown. Further studies are necessary to fully elucidate the complete pathway of temperature sensing and temperature-signal-transduction leading to temperature responses in plants.
